# Supplementary material for: Alpha-glucans from bacterial necromass indicate an intra-population loop within the marine carbon cycle
Source: Nat Commun. 2024 May 14;15:4048. doi: 10.1038/s41467-024-48301-5 (PMC11093988; doi:10.1038/s41467-024-48301-5)
Supplement: Supplementary file 1 — Supplementary Information [file 41467_2024_48301_MOESM1_ESM.pdf]

Supplementary information

Alpha-glucans from bacterial necromass indicate an intra-population loop within the marine carbon cycle

Irena Beidler[1], Nicola Steinke[2,3], Tim Schulze[1], Chandni Sidhu[2], Daniel Bartosik[1,4], Marie-Katherin Zühlke[1], Laura Torres Martin[1], Joris Krull[1,2], Theresa Dutschei[5], Borja Ferrero-Bordera[6], Julia Rielicke[6], Vaikhari Kale[6], Thomas Sura[6], Anke Trautwein-Schult[6], Inga V. Kirstein[7], Karen H. Wiltshire[7], Hanno Teeling[2], Dörte Becher[6], Mia Maria Bengtsson[8], Jan-Hendrik Hehemann[2,3], Uwe. T. Bornscheuer[5], Rudolf I. Amann[2], Thomas Schweder[1,4,7*]

[1] Pharmaceutical Biotechnology, Institute of Pharmacy, University of Greifswald, 17489 Greifswald, Germany

[2] Max Planck Institute for Marine Microbiology, 28359 Bremen, Germany

[3] University of Bremen, Center for Marine Environmental Sciences, MARUM, 28359 Bremen, Germany

[4] Institute of Marine Biotechnology, 17489 Greifswald, Germany

[5] Biotechnology and Enzyme Catalysis, Institute of Biochemistry, University of Greifswald, 17489 Greifswald, Germany

[6] Microbial Proteomics, Institute of Microbiology, University of Greifswald, 17489 Greifswald, Germany

[7] Alfred Wegener Institute for Polar and Marine Research, Biologische Anstalt Helgoland, 27483, Helgoland, Germany

[8] Microbial Physiology and Molecular Biology, Institute of Microbiology, University of Greifswald, 17489 Greifswald, Germany

[*] corresponding author:
Thomas Schweder, schweder@uni-greifswald.de

Supplementary figures

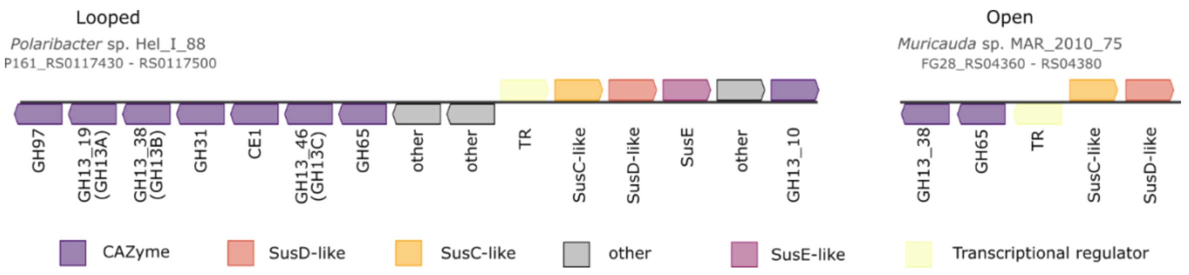

Figure S1. α-glucan PUL composition of *Polaribacter* sp. Hel_I_88 and *Muricauda* sp. MAR_2010_75. PULs contain at least one GH13 and the characteristic *susC/D* coding for the binding and uptake machinery. Complexity of the PUL seems to correspond to the type of SusD (detailed in **Figure 2**) encoded, with “looped” SusD-containing PULs encoding more CAZymes, while “open” SusD-containing PULs are simpler.

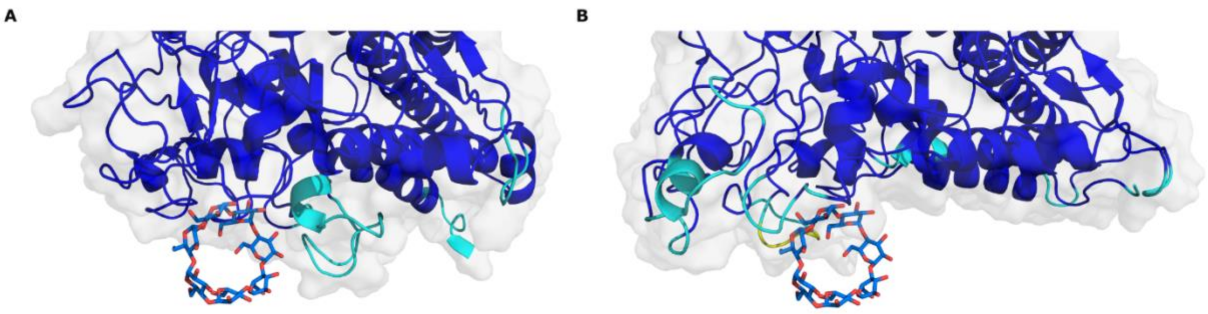

Figure S2. Structure prediction of the α-glucan PUL encoded SusDs. Shown is a surface model of (A) *Flavimarina* sp. Hel_I_48 (representative of the looped group, Sup. Dataset 5) and (B) *Muricauda* sp. MAR_2010_75 (representative of the open group, Sup. Dataset 6) alongside the structure cartoon overlayed with a cyclodextrin substrate (blue) as observed in *B. thetaiotaomicron* (PBD: 3CK8). Color of the models indicates the alpha fold confidence score (pLDDT) in the groups “very high” (>90) dark blue, “high” (90-71) cyan, “low” (70-51) yellow and “very low” (<50) orange.

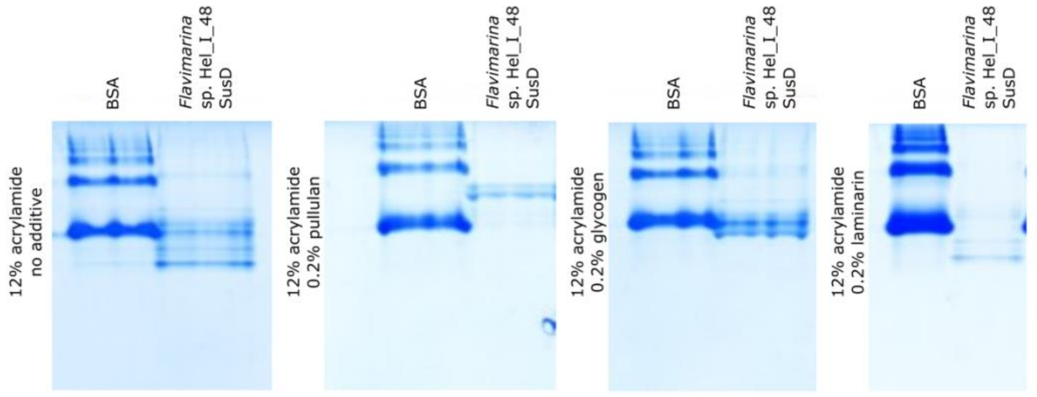

Figure S3. The α-glucan PUL-encoded SusD of *Flavimarina* sp. Hel_I_48 shows affinity to glycogen and pullulan. Experiments were performed using native gels with no additive or 0.2% polysaccharide. BSA was used as non-binding control.

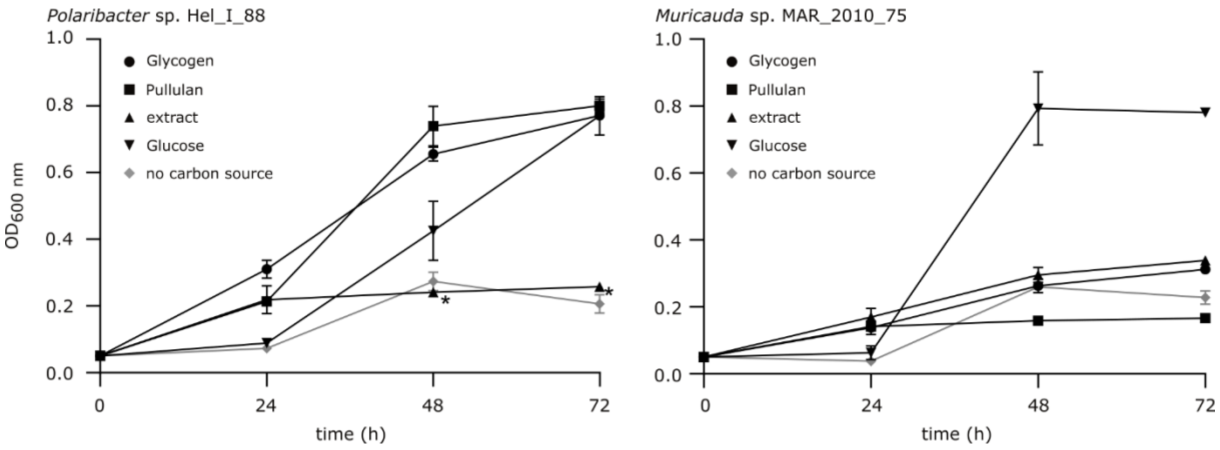

Figure S4. Growth of *Polaribacter* sp. Hel_I_88 and *Muricauda* sp. MAR_2020_75 on glycogen, pullulan and extracted intracellular polysaccharide of *Polaribacter* sp. Growth was determined (n=3) in marine minimal medium (MPM) containing the tested polysaccharide or extract as sole carbon source compared to controls using either glucose or no carbon source. *indicates the formation of bacterial aggregates during growth, hindering an accurate measurement.

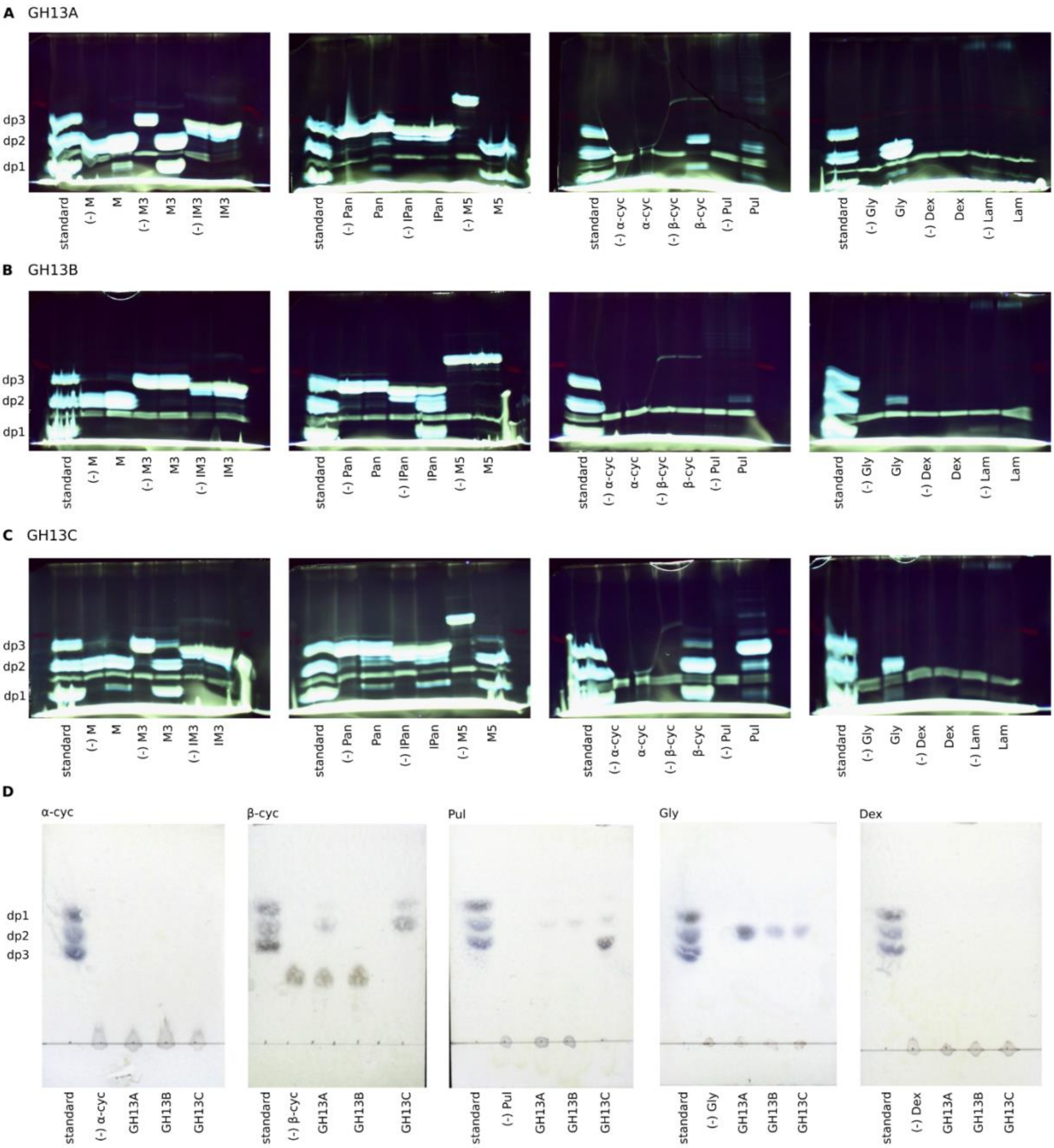

Figure S5. GH13 enzymes from *Polaribacter* sp. PUL act on diverse glucans. Degradation profiles of (**A**) GH13A, (**B**) GH13B and (**C**) GH13C via FACE on different poly- and oligosaccharides. (**D**) TLC-analysis of cylcodextrins and polysaccharides incubated with GH13A, GH13B and GH13C. Reactions were carried out with 25 μg of protein and 0.5% poly-/oligosaccharide for 24 h. M: Maltose, M3: Maltotriose, IM3: Isomaltotriose, Pan: Panose, IPan: Isopanose, M5: maltopentose α-cyc: α-cyclodextrin, β-cyc: β-cyclodextrin, Pul: Pullulan, Gly: Glycogen, Dex: Dextrin, Lam: Laminarin, (-): no enzyme.

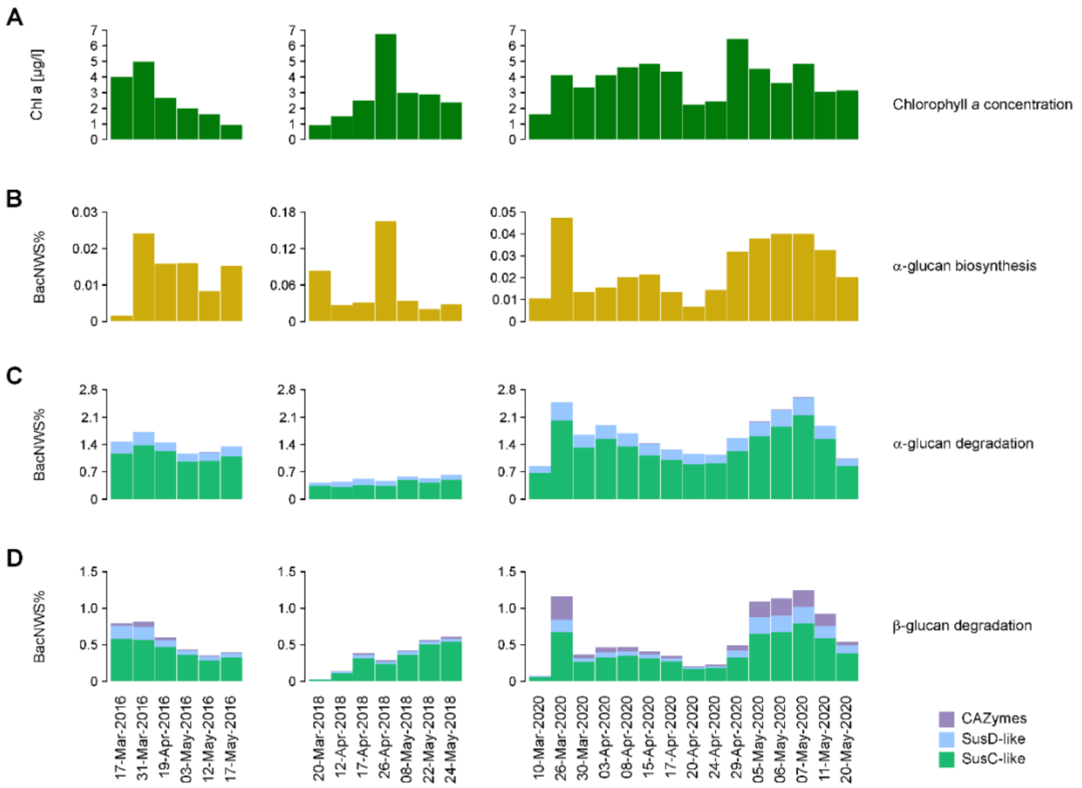

Figure S6. Glg-protein abundance during phytoplankton blooms relates to those of α-glucan and β-glucan-targeting proteins of the planktonic bacteria. (**A**) Progression of three sperate Helgoland bloom events (2016, 2018, 2020) as observed via chlorophyll *a* measurements. For each bloom, metaproteomes of the >0.2 µm fraction were searched for proteins involved in α-glucan biosynthesis (Glg-pathway) (**B**), α-glucan PUL (**C**) and β-glucan PUL-encoded (**D**) proteins. Relative abundances are given as normalized weighted spectra of the bacterial fraction (BacNWS%).

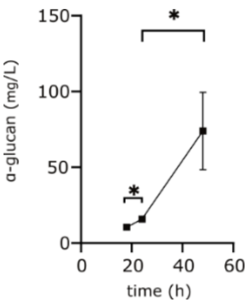

Figure S7. Bacteria produce significant amounts of α-glucan while growing on laminarin. Accumulated α-glucan amounts from three biological replicates of *Polaribacter* sp. Hel_I_88 grown on laminarin as sole carbon source (* = unpaired one-sided t-test P value < 0.05) as determined via specific enzymatic hydrolysis followed by PAHBAH-assay.

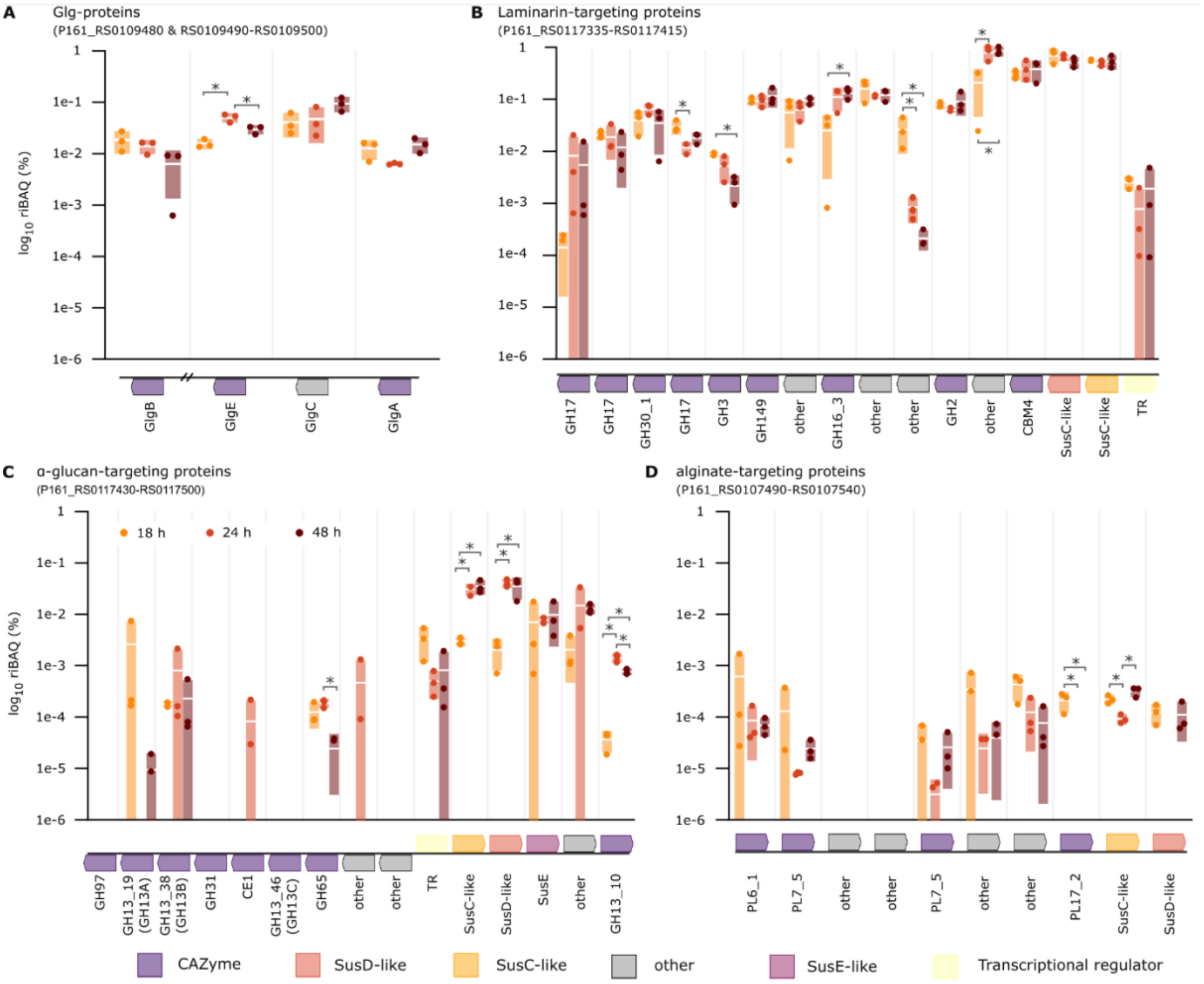

Figure S8. During growth on laminarin, *Polaribacter* sp. shows expression of proteins responsible for α-glucan synthesis and utilization. *Polaribacter* sp. was grown on laminarin as sole carbon source and samples were taken at 18, 24 and 48 h, respectively. Protein abundances of Glg-proteins (**A**), laminarin PUL proteins (**B**), α-glucan PUL proteins (**C**) and alginate PUL proteins (**D**) are shown over time. Given are all non-0 values with standard deviation (bars) and mean value (white line). Significance as determined by one-way ANOVA followed by post hoc Tukey's HSD test (*p*-value <0.05) is depicted as an asterisk with the bars. Abundances are given as manually calculated riBAQ (%) values. Gene clusters are depicted beneath the protein abundances and specific CAZyme annotations are provided underneath.

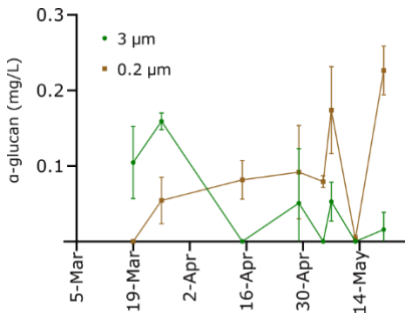

Figure S9. Bacteria of the 2020 Helgoland phytoplankton bloom produce α-glucan during peak bloom phases. α-glucan concentration on 0.2 µm and 3 µm fraction 2020 Helgoland bloom filters (three technical replicates) were determined via specific enzymatic hydrolysis followed by PAHBAH-assay. All values are corrected for volume filtered.

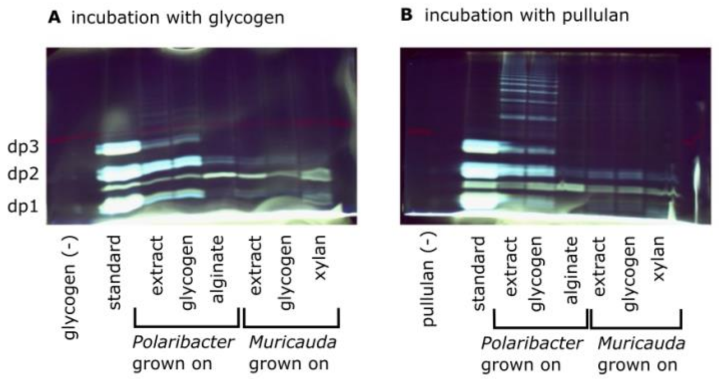

Figure S10. FACE-based enzyme activity assays of culture lysates of the two model bacteria *Polaribacter* sp. Hel_I_88 and *Muricauda* sp. MAR_2010_75 grown with different polysaccharides. Lysate of cultures grown on extracted bacterial α-glucans, glycogen and alginate or xylan were incubated (**A**) with glycogen and (**B**) with pullulan to test for substrate-induced differential PUL expression activities. Mixtures of glucose (dp1), maltose (dp2) and maltotriose (dp3) were used as standards.
