# Supplementary material for: Alpha-glucans from bacterial necromass indicate an intra-population loop within the marine carbon cycle
Source: Nat Commun. 2024 May 14;15:4048. doi: 10.1038/s41467-024-48301-5 (PMC11093988; doi:10.1038/s41467-024-48301-5)
Supplement: Supplementary file 3 — Description of Additional Supplementary Files [file 41467_2024_48301_MOESM3_ESM.pdf]

Description of Additional Supplementary Files:

Supplementary Dataset 1: 18S rDNA read counts of the Helgoland spring phytoplankton bloom from March to May 2020. Read counts from 3 and 10 µm fractions (Sheets 1 & 2) were summed. Relative abundances of groups of significant eukaryotes were plotted in Figure 1.

Supplementary Dataset 2: List of all isolateand MAG-encoded PUL-associated GH13s.

Supplementary Dataset 3: Proteomics of Polaribacter sp. Hel_I_88 grown on laminarin with samples taken at 18, 24 and 48 h

Supplementary Dataset 4: Proteomics of Polaribacter sp. Hel_I_88 and Muricauda sp. MAR_2010_75 grown on extracted polysaccharides from Polaribacter sp. as well as glycogen, alginate and xylan as controls.

Supplementary Data 5: Dataset for protein structure prediction of Flavimarina_Hel_1_48_SusD

Supplementary Data 6: Data set for protein structure prediction of Muricauda_MAR_2010_75_SusD
