# Supplementary material for: Alpha-glucans from bacterial necromass indicate an intra-population loop within the marine carbon cycle
Source: Nat Commun. 2024 May 14;15:4048. doi: 10.1038/s41467-024-48301-5 (PMC11093988; doi:10.1038/s41467-024-48301-5)
Supplement: Supplementary file 10 — Reporting Summary [file 41467_2024_48301_MOESM10_ESM.pdf]

nature portfolio

Corresponding author(s): Prof. Dr. Thomas Schweder

Last updated by author(s): Mar 26, 2024

Reporting Summary

Nature Portfolio wishes to improve the reproducibility of the work that we publish. This form provides structure for consistency and transparency in reporting. For further information on Nature Portfolio policies, see our Editorial Policies and the Editorial Policy Checklist.

Statistics

For all statistical analyses, confirm that the following items are present in the figure legend, table legend, main text, or Methods section.

n/a	Confirmed	
[ ]	[x]	The exact sample size (*n*) for each experimental group/condition, given as a discrete number and unit of measurement
[ ]	[x]	A statement on whether measurements were taken from distinct samples or whether the same sample was measured repeatedly
[ ]	[x]	The statistical test(s) used AND whether they are one- or two-sided<br>*Only common tests should be described solely by name; describe more complex techniques in the Methods section.*
[x]	[ ]	A description of all covariates tested
[ ]	[x]	A description of any assumptions or corrections, such as tests of normality and adjustment for multiple comparisons
[ ]	[x]	A full description of the statistical parameters including central tendency (e.g. means) or other basic estimates (e.g. regression coefficient) AND variation (e.g. standard deviation) or associated estimates of uncertainty (e.g. confidence intervals)
[ ]	[x]	For null hypothesis testing, the test statistic (e.g. *F*, *t*, *r*) with confidence intervals, effect sizes, degrees of freedom and *P* value noted<br>*Give P values as exact values whenever suitable.*
[ ]	[x]	For Bayesian analysis, information on the choice of priors and Markov chain Monte Carlo settings
[ ]	[x]	For hierarchical and complex designs, identification of the appropriate level for tests and full reporting of outcomes
[x]	[ ]	Estimates of effect sizes (e.g. Cohen's *d*, Pearson's *r*), indicating how they were calculated

Our web collection on statistics for biologists contains articles on many of the points above.

Software and code

Policy information about availability of computer code

Data collection	Unicorn v.7, Tecan iControl for infinite200 pro
Data analysis	ClustalO (v2.1),Unipro UGENE (v47), iTOL (v6.8.1), DADA2 (v1.26), SqueezeMeta v1.3.1, FragGeneScan v1.31, GenBank r239, eggNOG v5.0, KEGG r58.0, CAZy (as of 11/12/2023), Diamond v0.9.24.125, Pfam 33.0, HMMER3, Bowtie2, Mascot v2.7.0.1, Scaffold 4.11.1 & 5.0.1, X! Tandem v2017.2.1.4, GhostKOALA v2.0, hmmscan v3.3.2, KofamScan, MaxQuant,

For manuscripts utilizing custom algorithms or software that are central to the research but not yet described in published literature, software must be made available to editors and reviewers. We strongly encourage code deposition in a community repository (e.g. GitHub). See the Nature Portfolio guidelines for submitting code & software for further information.

Data

Policy information about availability of data

All manuscripts must include a data availability statement. This statement should provide the following information, where applicable:

- Accession codes, unique identifiers, or web links for publicly available datasets
- A description of any restrictions on data availability
- For clinical datasets or third party data, please ensure that the statement adheres to our policy

Metagenome, metatranscriptome and MAG sequence data are available from the European Nucleotide Archive (accession PRJEB52999; https://www.ebi.ac.uk/ena/browser/view/PRJEB52999). The mass spectrometry proteomic data have been deposited to the ProteomeXchange Consortium (http://proteomecentral.proteomexchange.org) via the PRIDE partner repository83 with the dataset identifier PXD019294 (bloom 2016; http://

proteomecentral.proteomexchange.org/cgi/GetDataset?ID=PXD019294), PXD042676 (bloom 2018; http://proteomecentral.proteomexchange.org/cgi/GetDataset?ID=PXD042676), PXD042805 (bloom 2020; http://proteomecentral.proteomexchange.org/cgi/GetDataset?ID=PXD042805), PXD043390 (Single strain proteomics; http://proteomecentral.proteomexchange.org/cgi/GetDataset?ID= PXD043390). All other data supporting the findings of this study are available within the paper and its Supplementary Information and source data is provided with the paper.

Research involving human participants, their data, or biological material

Policy information about studies with human participants or human data. See also policy information about sex, gender (identity/presentation), and sexual orientation and race, ethnicity and racism.

Reporting on sex and gender	na
Reporting on race, ethnicity, or other socially relevant groupings	na
Population characteristics	na
Recruitment	na
Ethics oversight	na

Note that full information on the approval of the study protocol must also be provided in the manuscript.

Field-specific reporting

Please select the one below that is the best fit for your research. If you are not sure, read the appropriate sections before making your selection.

☐ Life sciences ☐ Behavioural & social sciences ☒ Ecological, evolutionary & environmental sciences

For a reference copy of the document with all sections, see nature.com/documents/nr-reporting-summary-flat.pdf

Ecological, evolutionary & environmental sciences study design

All studies must disclose on these points even when the disclosure is negative.

Study description	Investigation of the microbiome of seasonal spring phytoplankton blooms in the North Sea.
Research sample	Microbial biomass from surface water in the North Sea, representing the microbiome of the spring phytoplankton bloom around the island of Helgoland in the North Sea.
Sampling strategy	Sampled surface water was sequentially filtered onto polycarbonate membrane filters with different pore sizes (10 micro m, 3 micro m and 0.2 mircro m); different pore sizes largely account for larger eukaryotes (10 micro m), smaller eukaryotes and attached bacteria (3 micro m) and free-living bacteria (0.2 mircro m)
Data collection	Metagenome, metatranscriptome, metaproteome, 16S rRNA, 18S rRNA, glycans
Timing and spatial scale	Subsurface seawater (1 m depth) was collected at 52 time points between 2nd of March and 26th of May 2020.
Data exclusions	Exclusion of Metazoan reads from 18S data sets as detailed in methods to recieve an accurate depiction of relevant microalgae during the bloom
Reproducibility	We used in all experiments at least three biological replicates.
Randomization	not relevant, sample collection from surface water
Blinding	not relevant, sample collection from surface water

Did the study involve field work? ☒ Yes ☐ No

Field work, collection and transport

Field conditions	Subsurface seawater (1 m depth) was collected at 52 time points between 2nd of March and 26th of May 2020 at the station Helgoland Roads near Helgoland in the southern North Sea. Since 1962 bucket water samples have been taken as part of a long-term monitoring program Helgoland Roads (54°11'N 7°54'E; DEIMS.iD: https://deims.org/1e96ef9b-0915-4661-849f-b3a72f5aa9b1)41.
Location	Helgoland Roads (54°11'N 7°54'E)
Access & import/export	Since 1962 bucket water samples have been taken as part of a long-term monitoring program Helgoland Roads. Membran filter

	samples were frozen and stored at -80 °C.
Disturbance	no

Reporting for specific materials, systems and methods

We require information from authors about some types of materials, experimental systems and methods used in many studies. Here, indicate whether each material, system or method listed is relevant to your study. If you are not sure if a list item applies to your research, read the appropriate section before selecting a response.

Materials & experimental systems

n/a	Involved in the study
☒	☐ Antibodies
☒	☐ Eukaryotic cell lines
☒	☐ Palaeontology and archaeology
☒	☐ Animals and other organisms
☒	☐ Clinical data
☒	☐ Dual use research of concern
☒	☐ Plants

Methods

n/a	Involved in the study
☒	☐ ChIP-seq
☒	☐ Flow cytometry
☒	☐ MRI-based neuroimaging

Plants

Seed stocks	na
Novel plant genotypes	na
Authentication	na
