# Supplementary material for: Alpha-glucans from bacterial necromass indicate an intra-population loop within the marine carbon cycle
Source: Nat Commun. 2024 May 14;15:4048. doi: 10.1038/s41467-024-48301-5 (PMC11093988; doi:10.1038/s41467-024-48301-5)
Supplement: Supplementary file 11 — Source data [file 41467_2024_48301_MOESM11_ESM.zip › Source_data/Source_data/Source_data_blots.pptx]

## Slide 1
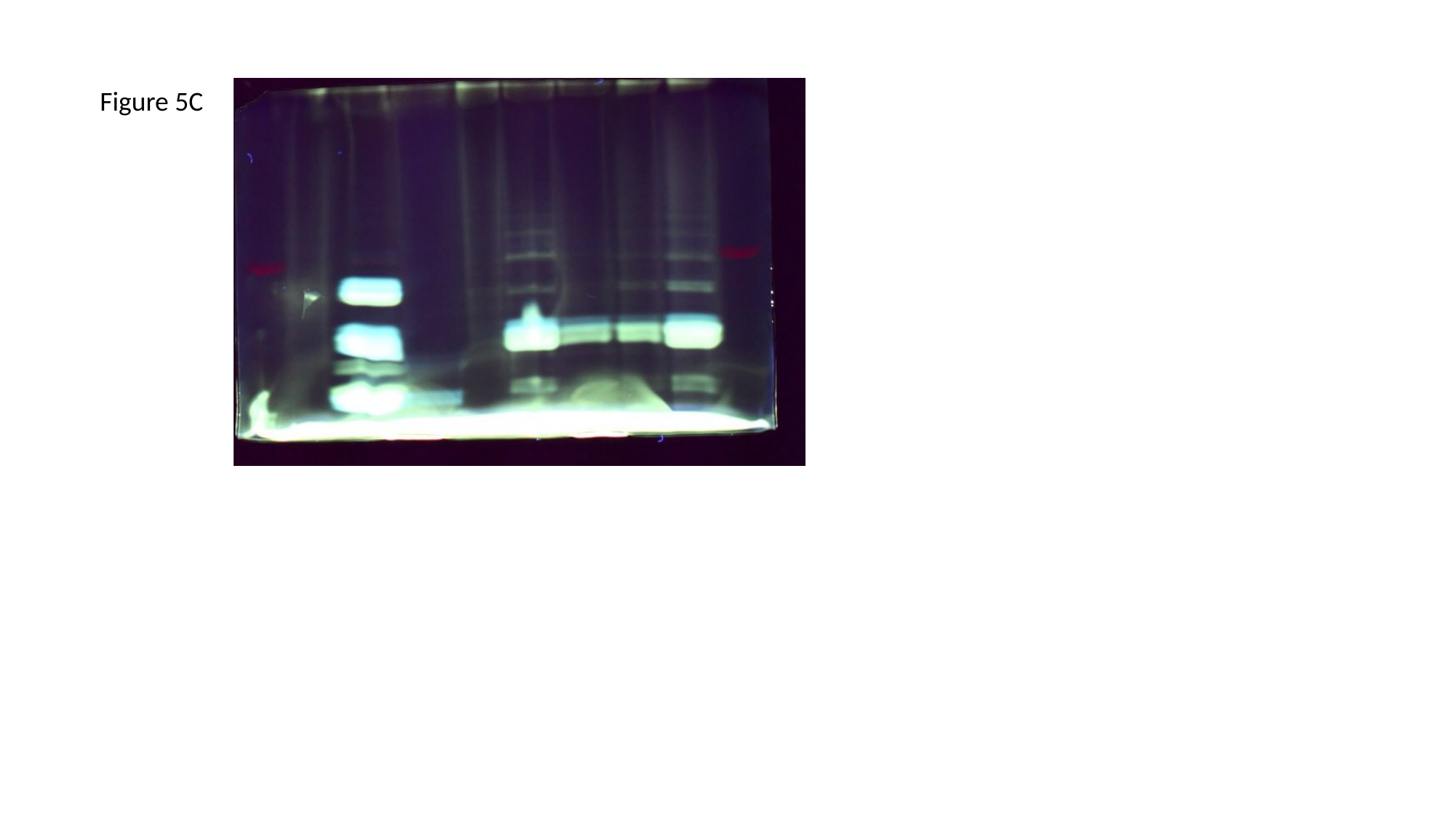

Figure 5C

## Slide 2
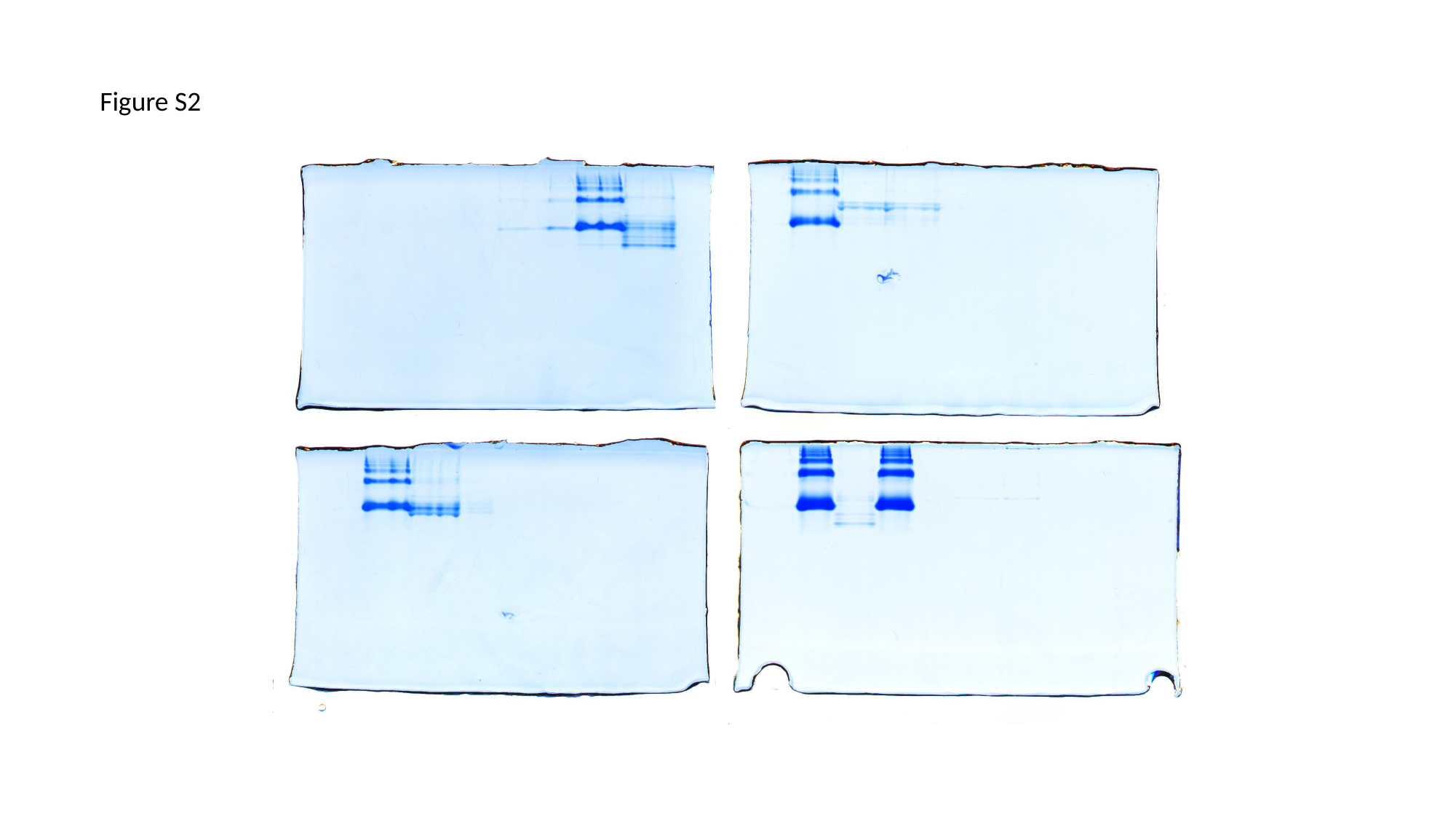

Figure S2

## Slide 3
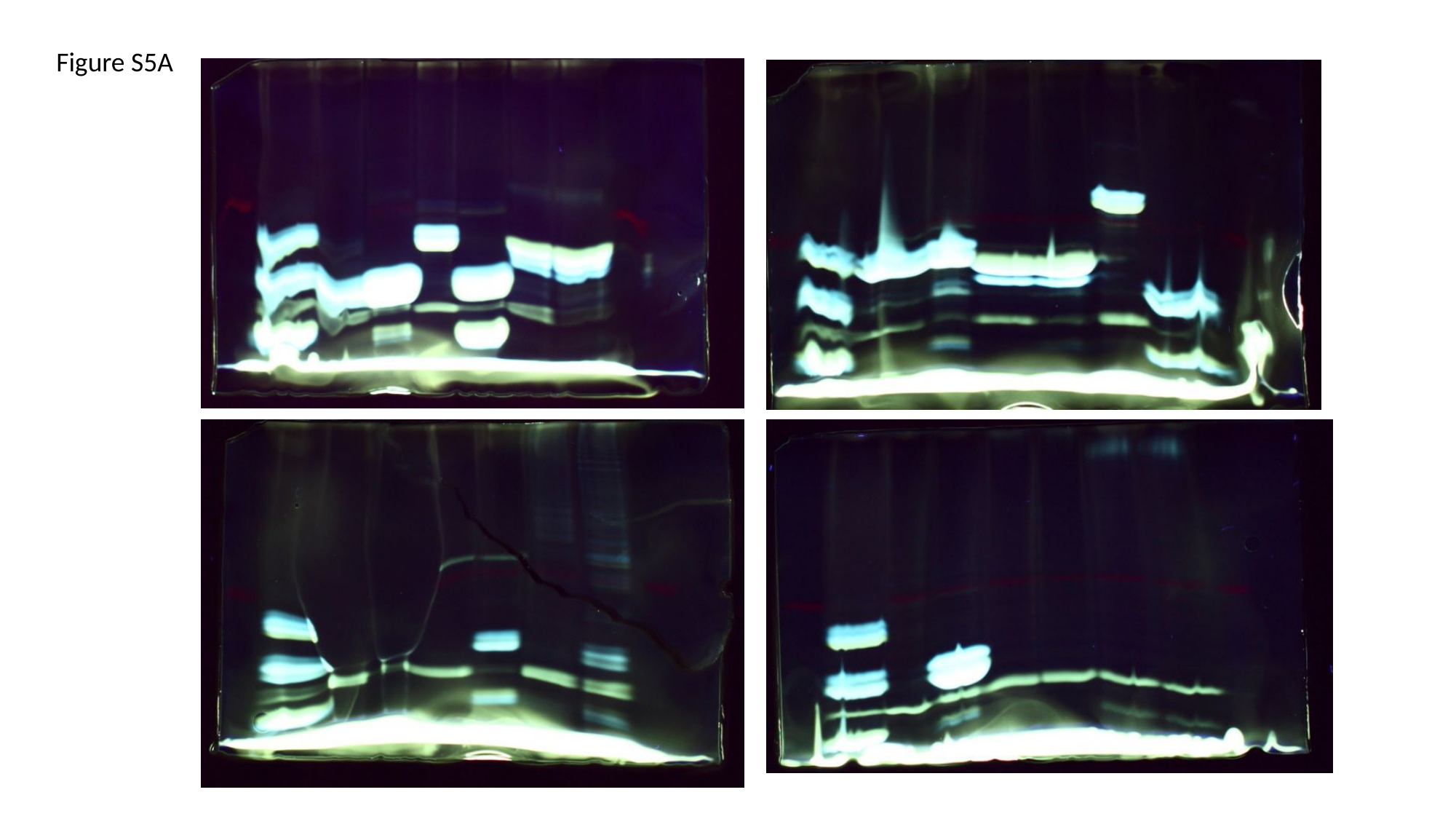

Figure S5A

## Slide 4
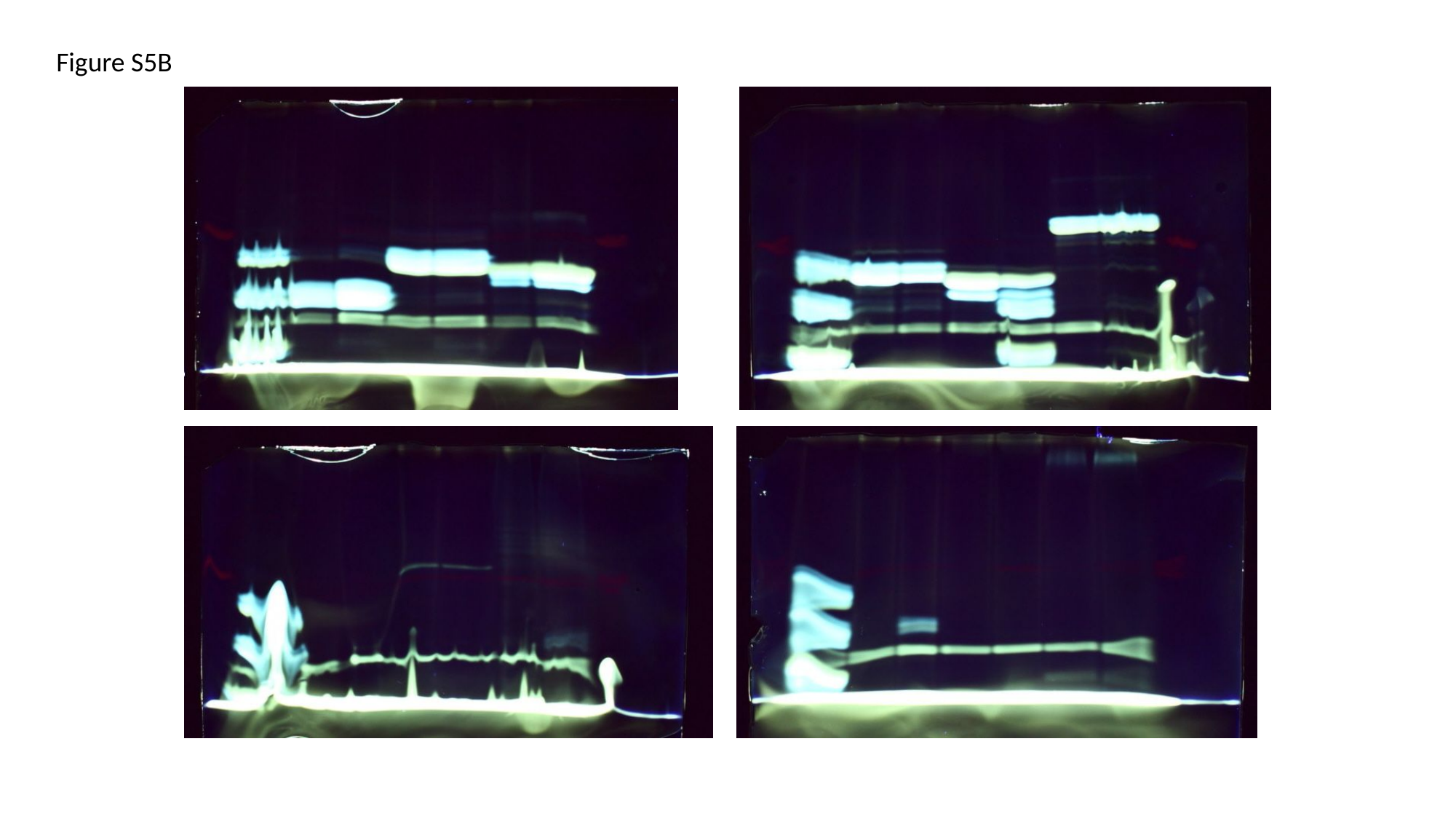

Figure S5B

## Slide 5
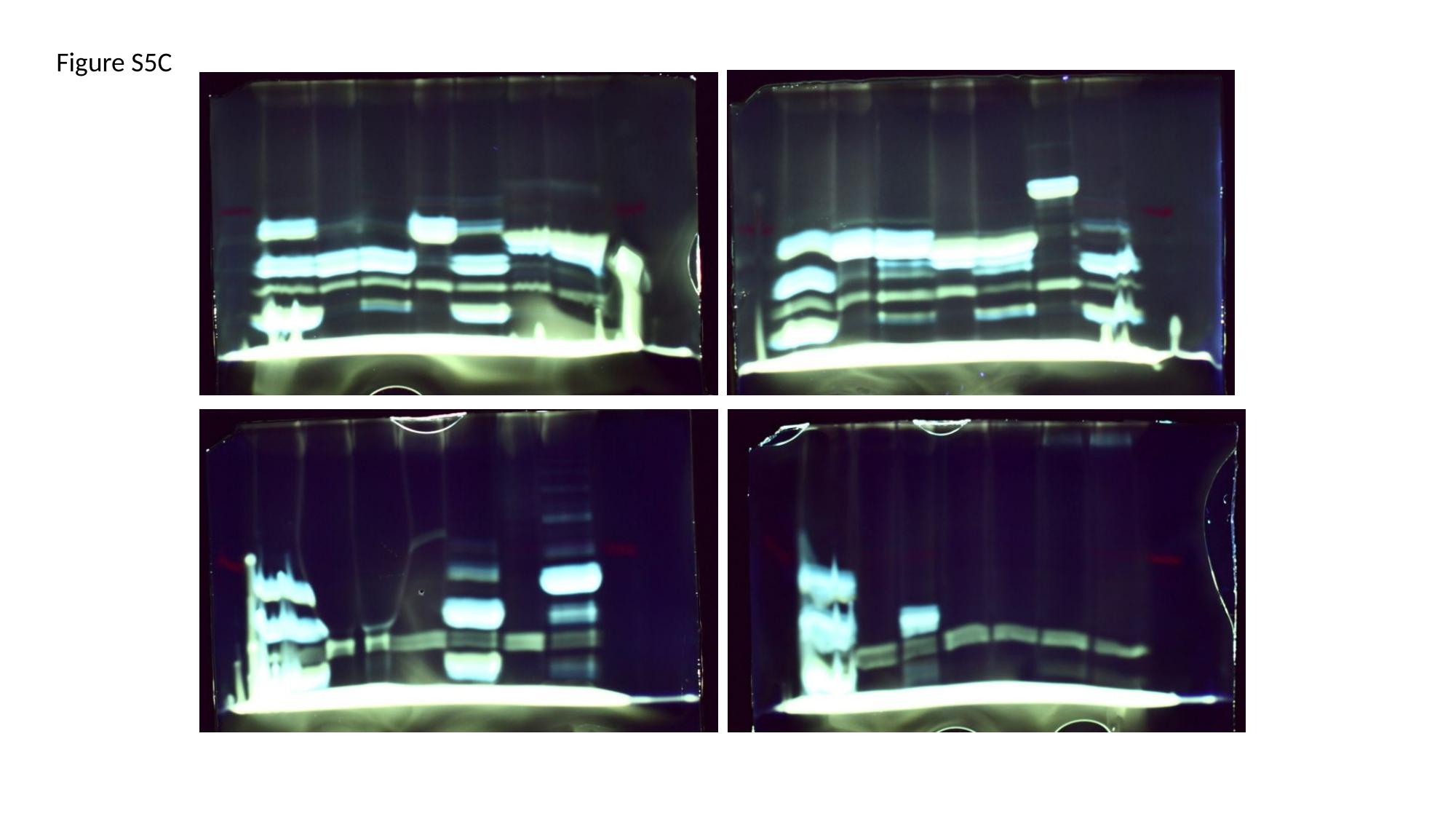

Figure S5C

## Slide 6
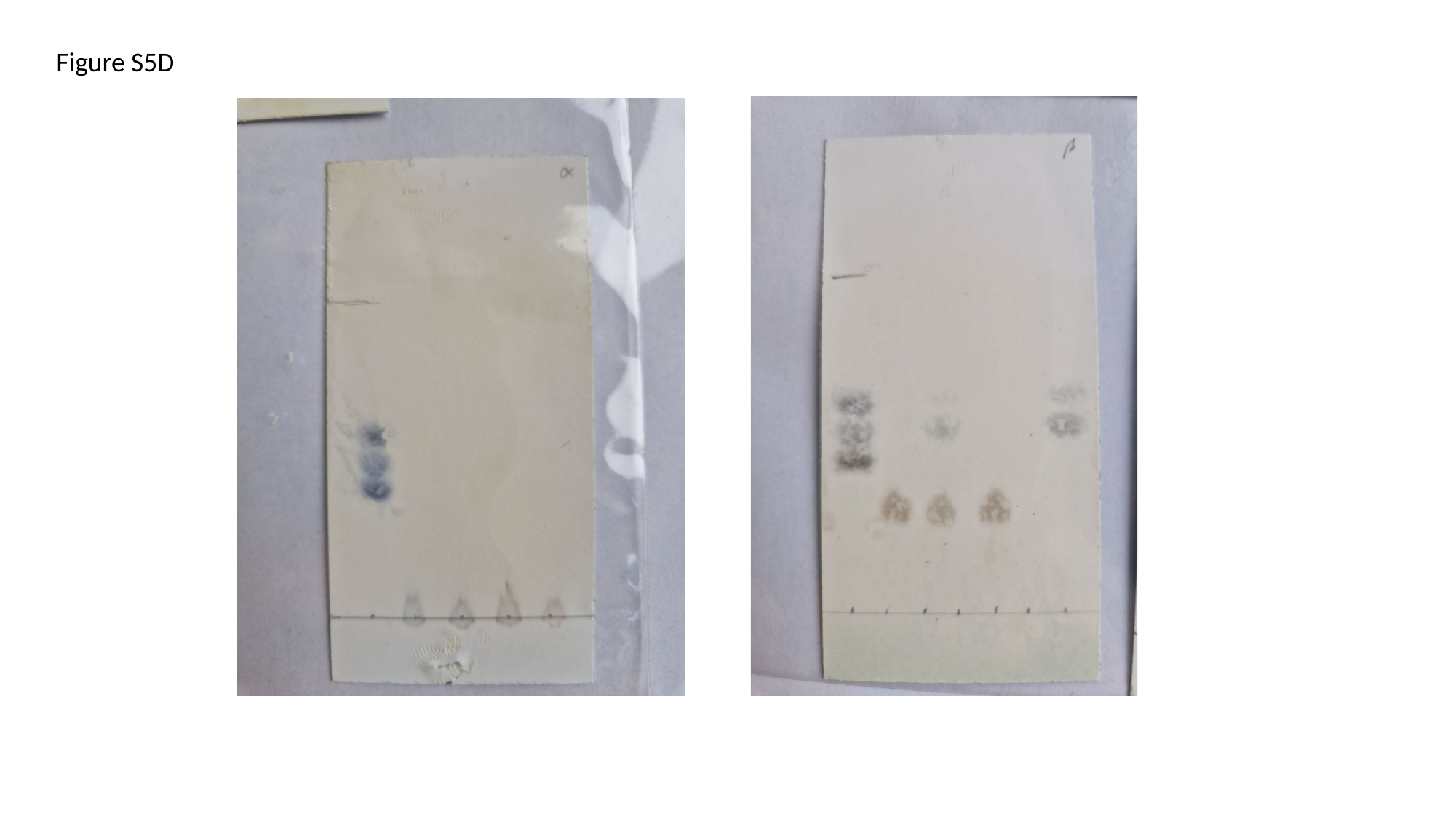

Figure S5D

## Slide 7
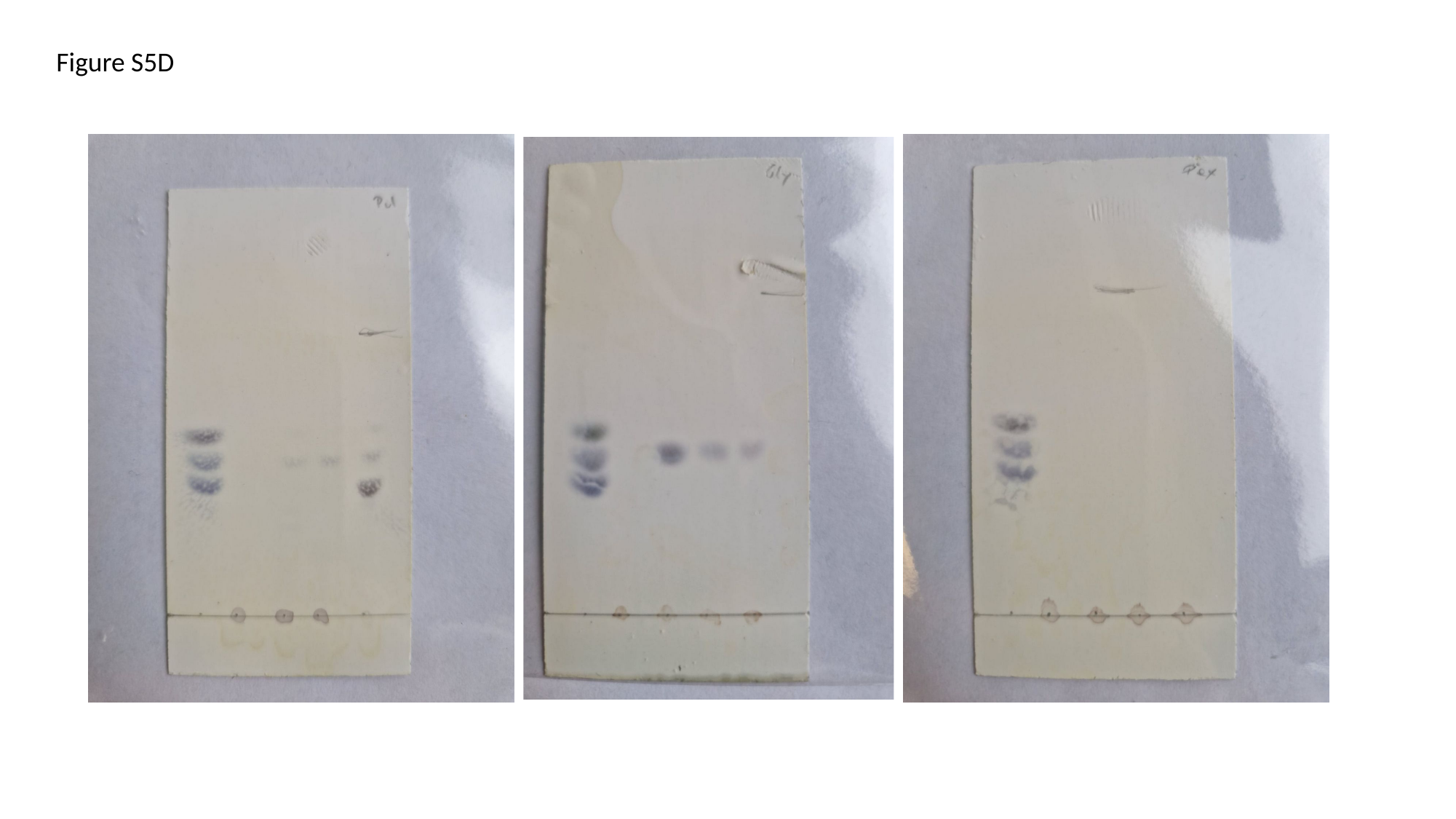

Figure S5D

## Slide 8
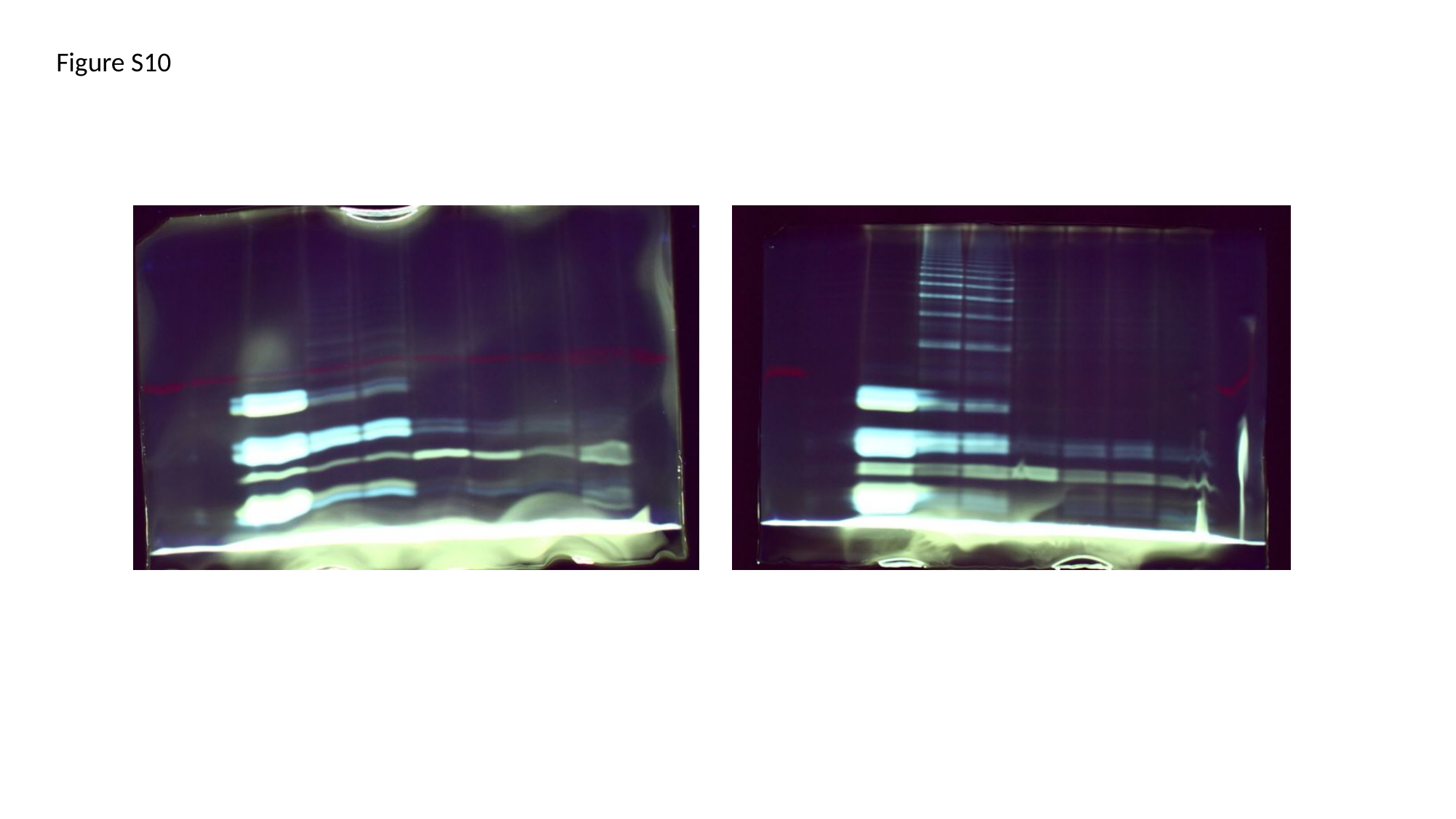

Figure S10
